# Longitudinal Association between Serum Leptin Concentration and Glomerular Filtration Rate in Humans

**DOI:** 10.1371/journal.pone.0117828

**Published:** 2015-02-24

**Authors:** Claudio Pedone, Baback Roshanravan, Simone Scarlata, Kushang V. Patel, Luigi Ferrucci, Raffaele Antonelli Incalzi

**Affiliations:** 1 Unit of Geriatrics, “Campus Biomedico” University, Rome, Italy; 2 Department of Medicine, Division of Nephrology, University of Washington—Kidney Research Institute, Seattle, Washington, United States of America; 3 Department of Anesthesiology and Pain Medicine, University of Washington, Seattle, Washington, United States of America; 4 Longitudinal Study Section, Clinical research Branch, National Institute of Aging, Baltimore, Maryland, United States of America; 5 “S. Raffaele—Cittadella della Carità” Foundation, Taranto, Italy; Ichan School of Medicine at Mount Sinai, UNITED STATES

## Abstract

**Background:**

Obesity is a risk factor for decline in glomerular filtration rate (GFR). One proposed mechanism leading to glomerulopathy is an increase in leptin levels. However, the association between leptin and GFR has never been demonstrated. The aim of this study is to verify whether higher levels of leptin are associated with longitudinal changes of estimated GFR (eGFR).

**Methods and findings:**

We selected 744 participants in the InCHIANTI study (416 women). The association between eGFR and leptin changes over a 6-years follow-up was assessed using random effect models including leptin as a time-varying covariate and adjusted for potential confounders. We also compared the proportion of patients with rapid decline of renal function across tertiles of change in serum leptin between baseline and 6-years follow-up. Mean baseline eGFR was 82.2 ml/min/1.73 m, 78.7 ml/min/1.73 m, and 75.4 ml/min/1.73 m in the first, second and third tertile of baseline serum leptin concentration, respectively. After adjustment for potential confounders, leptin concentration was inversely associated with changes of eGFR over time (β for log-leptin: -1.288, 95% CI: -2.079 – -0.497). Relative to baseline levels, the estimated change in eGFR for unit-increase in log-leptin was -1.9% (95% CI: -2.977 – -0.761). After stratification by sex, the results were confirmed in women only. In women we also found an association between increasing leptin concentration over time and rapid decline of renal function.

**Conclusions:**

In women, serum leptin may contribute to eGFR decline independently from obesity and diabetes mellitus, although a cause-effect relationship cannot be established due to the observational nature of our study. A better characterization of adipokine profile of obese individuals may shed light on the accelerated renal function decline reported in a proportion of high-risk obese individuals.

## Introduction

Obesity is traditionally considered a risk factor for chronic kidney disease (CKD) and end-stage renal disease (ESRD) both independently and due to its link with diabetes mellitus and hypertension. Evidence from large retrospective cohort studies shows an association of obesity and risk of CKD [1–6] and ESRD [5]. Higher baseline body mass index (BMI) among participants in the Hypertension Detection and Follow-up Program has been independently associated with higher 5-year incidence of CKD [7]. Results from other cohorts confirm that obesity per se affects renal function [8, 9]. Two potential mechanism underlying impaired renal function in obesity are glomerular hyperfiltration and leptin-mediated glomerular injury.

Glomerular hyperfiltration [10, 11], increased renin-angiotensin activity and sympathetic tone as well as enhanced sodium reabsorption [12, 13] have been well-described in obese individuals. Hyperfiltration, in turn, causes microalbuminuria by damaging the filtration barrier. Indeed, different indexes of overweight (BMI, waist to hip ratio and waist circumference) have been associated with increased albuminuria in both non-diabetic and diabetic populations [14–17]. Weight reduction has also been demonstrated to reduce albuminuria in non-diabetic obese individuals [18, 19].

Adiposity has been associated with increased levels of circulating inflammatory cytokines even in the absence of diabetes [20] and obesity-related glomerulopathy has been shown to have a distinct pathological picture [21]. In particular, visceral adiposity has been demonstrated to have a negative effect on the kidney and is associated with glomerulopathy characterized by glomerulomegaly, podocyte injury, and segmental sclerosis. One potential mechanism for such pathology is a reduction in adiponectin and an increase in leptin. Adiponectin has been associated with important renoprotective effects in the animal models [22, 23], and has been found to be reduced in sera of obese patients [24]. Leptin serum concentration, instead, is typically increased in both obesity and CKD, and mice over-expressing leptin are at increased risk of CKD [25]. Furthermore, rats chronically given leptin infusion develop albuminuria and glomerulosclerosis [26].

We hypothesize that higher leptin levels are associated with GFR decline over time. The aim of this study is to describe the association of serum leptin concentration with longitudinal change in estimated glomerular filtration rate (eGFR) over a 6 year follow-up among participants enrolled in the InCHIANTI study.

## Methods

### Data source

We used data from the InCHIANTI study, which was designed to investigate the factors contributing to the decline of mobility in older persons [27]. The participants in the study were randomly selected from the populations of two town areas in the Chianti region: Greve in Chianti and Bagno a Ripoli. The Italian National Institute of Research and Care on Aging ethical committee ratified the study protocol. Participants received an extensive description of the study and signed an informed participation consent that included permission to conduct analysis on the biological specimens collected and stored. For those unable to fully consent because of cognitive or physical problems, surrogate consent was also obtained from a close relative. The eligible participants were interviewed at their homes by trained study researchers using a structured questionnaire aimed at investigating their health status, their physical and cognitive performance, and other factors possibly related to loss of independence in late life. The interview was followed by a physical examination at the study clinic. Participants were followed-up with evaluations at 3 and 6 years.

### Laboratory assays

Blood samples obtained after the patient had fasted for 12 hours and rested for at least 15 minutes were centrifuged and stored at -80 until analyzed. Serum leptin was determined using ELISA (Human Endocrine LINCOplex Kit; MDC = 1 ng/ml in 100 μl sample, CV <7%). Estimated glomerular filtration rate (eGFR) was calculated using the CKD-EPI formula [28].

### Analytic approach

Among 1217 patients with both serum creatinine and leptin measured at baseline, 744 persons who had creatinine measured at both the 3- and 6-year follow-up visits. Of those excluded, 213 had died before of the 6-years follow-up.

Since exploratory analysis showed that leptin had a skewed distribution, this variable was log-transformed obtaining a normal distribution (skewness: -0.3, kurtosis: 0.08). We described the characteristics of the study sample according to tertiles of baseline leptin, including in this analysis variables deemed to influence eGFR: age, sex, body mass index, smoking status (pack-years), lipid profile, inflammatory markers (high-sensitivity C-reactive protein—hsCRP, interleukin 6—IL6, tumor necrosis factor alpha—TNF-α), diagnosis of diabetes mellitus and hypertension. Pack-years and all laboratory variables except HDL and LDL were log-transformed to obtain a normal distribution. We included the waist-to-hip ratio as measure for central adiposity, as it is more strongly associated with eGFR decline than BMI [29]. We also included an indicator variable for level of physical activity in the last year (categorized as less than vs. at least moderate exercise 1 or 2 times/week or light exercise more than 4 times/week). Differences across tertiles were tested using analysis of variance for continuous variables and χ-square test for categorical variables.

To evaluate the longitudinal association between eGFR and leptin we used a mixed linear model with random intercept in which leptin was modeled as a time-dependent covariate. The model was adjusted for potential confounders using nested models. Model 1 included leptin, baseline age, sex, and waist-to-hip ratio; model 2 added smoking, systolic blood pressure, hypertension, diabetes, triglycerides, LDL-cholesterol, HDL-cholesterol, C-reactive protein, IL6, TNF and use of ACEI/ARB. All the variables were modeled as time-dependent covariates except baseline age and sex and use of ACEI/ARB; for these variables an interaction term with time was included. BMI was not included in the models to avoid collinearity with waist-to-hip ratio. To account for the role of baseline eGFR, the same models were evaluated using the log of eGFR as the dependent variable. The coefficients of such a model allow to calculate the expected subject-specific mean percent difference in eGFR using the following formula: (e*100)-100.

Sensitivity analyses were performed including all patients having at least 1 follow-up and excluding from the models variables that may lie in the causal pathway between leptin and reduced eGFR (hypertension, diabetes, systolic blood pressure, cholesterol, triglycerides).

As there is evidence that the relationship between adiposity and renal disease differs between men and women [30, 31], we decided a-priori to evaluate the multivariable models separately in men and women.

Finally, we examined the risk of rapid decline in renal function, defined as a loss of more than 3 ml/min/1.73 m2/year [32] according to tertiles of leptin change over follow-up time. The analyses were performed using R for Linux version 3.0.1.

## Results

### Sample characteristics

**Fig 1:**
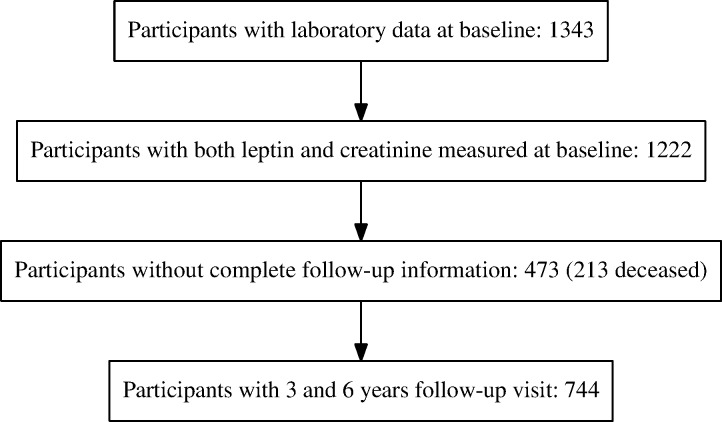
CONSORT diagram of the study.

We studied 744 patients who completed both the 3- and the 6-years follow-up (see Fig. 1 for a CONSORT diagram of the study). The mean age was 64.4 years (SD: 15.6), 55.9% of participants were women. Prevalence of hypertension and diabetes mellitus was 54.4% and 9.9%, respectively. Patients not included in the analysis were older (mean age: 74.3 years, SD: 14.1), had on average a lower baseline eGFR (70.2 ml/min/1.73 m compared to 78.8 ml/min/1.73 m), but presented similar serum leptin concentration and waist-to-hip ratio compared to those who were included. There was no difference in mean age across tertiles of leptin concentration, while women were more prevalent among patients in the third tertile (87% vs. 29% in the first tertile, P < 0.001). Mean baseline eGFR was 82.2 ml/min/1.73 m, 78.7 ml/min/1.73 m, and 75.4 ml/min/1.73 m in the first, second and third tertile of serum leptin concentration, respectively. As expected, leptin concentration was positively correlated with blood lipid concentration (Table 1).

**Table 1:** Distribution of the variables of interest by tertiles of baseline leptin.

	All (N: 744)	I tertile (N: 248)	II tertile (N: 248)	III tertile (N: 248)	P-value
Age, mean (SD)	64.4 (15.6)	63.5 (16.8)	64.3 (15.3)	65.5 (14.7)	0.352
Women, %	55.9	29	52	87	< 0.001
eGFR, mean(SD)	78.8 (16.1)	82.2 (16.2)	78.7 (15.9)	75.4 (15.4)	< 0.001
Waist to hip ratio, mean (SD)	0.9 (0.1)	0.9 (0.1)	0.9 (0.1)	0.9 (0.1)	0.001
Smoking (packyears), mean (SD)	10.4 (18.1)	13.5 (19.7)	12.6 (20.2)	5.1 (11.9)	< 0.001
Systolic blood pressure (mmHg), mean (SD)	146 (21.4)	144.4 (21.1)	147.6 (22.3)	146 (20.8)	0.252
Triglycerides (mg/dl), mean (SD)	123.3 (70.6)	114 (60.3)	130.9 (87.3)	125 (59.9)	0.025
Total cholesterol (mg/dl), mean (SD)	215.9 (40.7)	209.1 (39.6)	215.2 (40)	223.4 (41.4)	< 0.001
LDL cholesterol (mg/dl), mean (SD)	135.1 (36.3)	129.7 (35.3)	134.3 (35.8)	141.2 (36.9)	0.002
HDL cholesterol (mg/dl), mean (SD)	56.2 (14.7)	56.7 (14.6)	54.8 (15.1)	57.1 (14.3)	0.162
C-reactive protein (microg/dl), mean (SD)	4.1 (7.2)	3.2 (5.5)	4.3 (8.8)	4.9 (6.9)	0.034
Interleukin 6 (pg/ml), mean (SD)	3 (2.2)	3 (2.4)	3.1 (2.4)	3 (1.8)	0.664
Tumor necrosis factor alpha (pg/ml), mean (SD)	4.7 (8)	4.3 (1.9)	4.5 (3.6)	5.5 (13.3)	0.218
Hypertension (%)	54.4	52	56	55	0.734
Diabetes mellitus (%)	9.9	9	10	11	0.657
ACE-inhibitors (%)	1	1	1	3	0.1
AT-receptor blockers (%)	16	12	18	17	0.157
Diuretics (%)	5	2	6	8	0.015
Calcium-channel blockers (%)	12	11	13	11	0.814
NSAIDs (%)	6	4	5	9	0.092

### Association of longitudinal changes in leptin with change in eGFR

**Fig 2:**
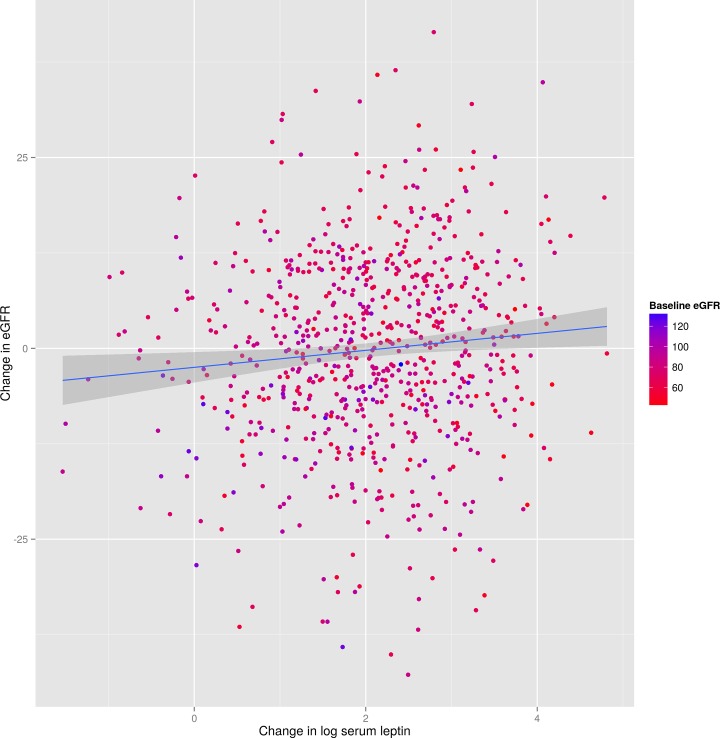
Relationship between changes in eGFR at 6 years and change in (log) leptin at 6 years. Colors indicate baseline eGFR values.

Increasing leptin concentration over follow-up time was associated with greater reduction of eGFR, without a noticeable effect of baseline eGFR (Fig. 2). The estimated reduction of eGFR for each 1-unit increase of log leptin adjusted for age, sex, waist-to-hip ratio and smoking exposure was -1.458 ml/min/1.73 m (95% CI: -2.204, -0.712) (Table 2). After adjustment for the other potential confounders (systolic blood pressure, lipids, indices of inflammation, and diagnosis of diabetes or hypertension) the estimate was -1.288 (95% CI: -2.079, -0.497) (see Table 2, second column). In this adjusted model, other variables associated with GFR changes were age (β: -0.741, 95% CI: -0.803, -0.679), systolic blood pressure (β: 0.064, 95% CI: 0.025, 0.103), and LDL cholesterol (β: -0.37, 95% CI: -3.607, 2.866). A sensitivity analysis including patients having at least 1 follow-up (N = 1217) yielded similar results (adjusted β for log leptin: -1.227; 95% CI: -1.921, -0.533). Removing from the model variables potentially lying on the causal pathway of the relationship between leptin and GFR decline, the β the effect of leptin became more evident (β: -1.534, 95% CI: -2.311, -0.757). When log eGFR was modeled, therefore taking into account baseline levels of eGFR, the estimated mean percent change in eGFR for each unit increase of leptin, back-transformed from the log scale to the natural scale, was -1.875% (95% CI: -2.977, -0.761).

**Table 2:** Mixed linear models modeling eGFR change.

	eGFR, unadjusted model	eGFR, adjusted model	log(eGFR), unadjusted model	log(eGFR), adjusted model
Log leptin	-1.458 (-2.204, -0.712)	-1.288 (-2.079, -0.497)	-2.069 (-3.112, -1.014)	-1.875 (-2.977, -0.761)
Age	-0.703 (-0.753, -0.652)	-0.741 (-0.803, -0.679)	-0.877 (-0.949, -0.804)	-0.93 (-1.019, -0.841)
Sex (women)	-3.146 (-5.377, -0.915)	-2.784 (-5.285, -0.283)	-4.129 (-7.155, -1.004)	-3.527 (-6.928, -0.002)
Waist to hip ratio	-1.118 (-10.984, 8.747)	-0.446 (-10.51, 9.617)	-1.537 (-14.46, 13.34)	-0.777 (-13.97, 14.44)
Smoking	-	-0.099 (-0.672, 0.474)	-	-0.038 (-0.867, 0.798)
Systolic blood pressure	-	0.064 (0.025, 0.103)	-	0.107 (0.051, 0.162)
Hypertension	-	2.566 (0.339, 4.792)	-	3.302 (0.051, 6.66)
Diabetes	-	-0.37 (-3.607, 2.866)	-	-0.908 (-5.41, 3.809)
Triglycerides	-	-1.493 (-3.102, 0.115)	-	-1.81 (-4.036, 0.469)
LDL cholesterol	-	-0.042 (-0.06, -0.024)	-	-0.058 (-0.084, -0.033)
HDL cholesterol	-	-0.019 (-0.071, 0.033)	-	-0.005 (-0.079, 0.069)
C-reactive protein	-	0.068 (-0.62, 0.757)	-	0.326 (-0.653, 1.315)
IL-6	-	1.037 (-0.076, 2.15)	-	1.271 (-0.315, 2.881)
TNF	-	-1.079 (-2.091, -0.066)	-	-1.693 (-3.098, -0.269)
Physical activity (high vs. low)	-	-0.133 (-1.405, 1.138)	-	0.113 (-1.672, 1.93)
Use of ACEI/ARB	-	-0.09 (-2.783, 2.604)	-	-0.557 (-4.334, 3.369)

Mean BMI was similar in men (26.9 kg/m) and women (27.2 kg/m), as was waist-to-hip ratio (0.94 in men and 0.87 in women). Leptin serum concentration, however, was higher in women (17.9, SD 8.1) than in men (6.7, SD: 15.6). The association between leptin and eGFR change was more evident in women (β: -1.664, 95% CI: -2.806, -0.523) than in men (β: -0.732, 95% CI: -1.836, 0.371). This difference was similar in the regressions modeling the log of eGFR (Table 3).

**Table 3:** Mixed linear models modeling eGFR change, by sex.

	eGFR, men	eGFR, women	log(eGFR), men	log(eGFR), women
Log leptin	-0.732 (-1.836, 0.371)	-1.664 (-2.806, -0.523)	-1.055 (-2.56, 0.474)	-2.47 (-4.074, -0.8398)
Age	-0.755 (-0.843, -0.666)	-0.716 (-0.806, -0.625)	-0.934 (-1.058, -0.81)	-0.904 (-1.036, -0.773)
Waist to hip ratio	3.744 (-12.66, 20.15)	-4.603 (-17.562, 8.356)	5.451 (-15.9, 32.222)	-6.083 (-22.1, 13.22)
Smoking	-0.436 (-1.173, 0.301)	0.436 (-0.529, 1.401)	-0.493 (-1.528, 0.554)	0.587 (-0.837, 2.031)
Systolic blood pressure	0.052 (-0.001, 0.106)	0.07 (0.014, 0.126)	0.084 (0.01, 0.157)	0.121 (0.04, 0.2017)
Hypertension	2.184 (-0.95, 5.318)	2.632 (-0.532, 5.796)	3.752 (-0.714, 8.419)	2.535 (-2.096, 7.3853)
Diabetes	-1.934 (-6.327, 2.459)	1.079 (-3.677, 5.834)	-3.083 (-8.881, 3.084)	1.204 (-5.589, 8.4862)
Triglycerides	-2.346 (-4.555, -0.137)	0.13 (-2.235, 2.494)	-2.476 (-5.421, 0.561)	0.121 (-3.259, 3.6201)
LDL cholesterol	-0.047 (-0.073, -0.021)	-0.042 (-0.066, -0.018)	-0.067 (-0.103, -0.03)	-0.056 (-0.091, -0.021)
HDL cholesterol	0.011 (-0.069, 0.091)	-0.026 (-0.095, 0.043)	0.047 (-0.065, 0.158)	-0.019 (-0.119, 0.0818)
C-reactive protein	0.469 (-0.51, 1.448)	-0.269 (-1.244, 0.706)	0.928 (-0.43, 2.305)	-0.131 (-1.535, 1.2922)
IL-6	0.71 (-0.923, 2.343)	1.302 (-0.218, 2.823)	0.5 (-1.74, 2.791)	1.812 (-0.395, 4.0681)
TNF	-0.568 (-1.908, 0.772)	-1.545 (-3.052, -0.038)	-0.642 (-2.465, 1.215)	-2.601 (-4.704, -0.451)
Physical activity (high vs. low)	-0.641 (-2.26, 0.978)	0.56 (-1.413, 2.533)	-0.568 (-2.759, 1.672)	1.127 (-1.705, 4.041)
Use of ACEI/ARB	-0.401 (-4.501, 3.699)	0.081 (-3.511, 3.674)	-0.627 (-6.192, 5.268)	-0.428 (-5.521, 4.94)

### Association of leptin with rapid kidney function decline

**Fig 3:**
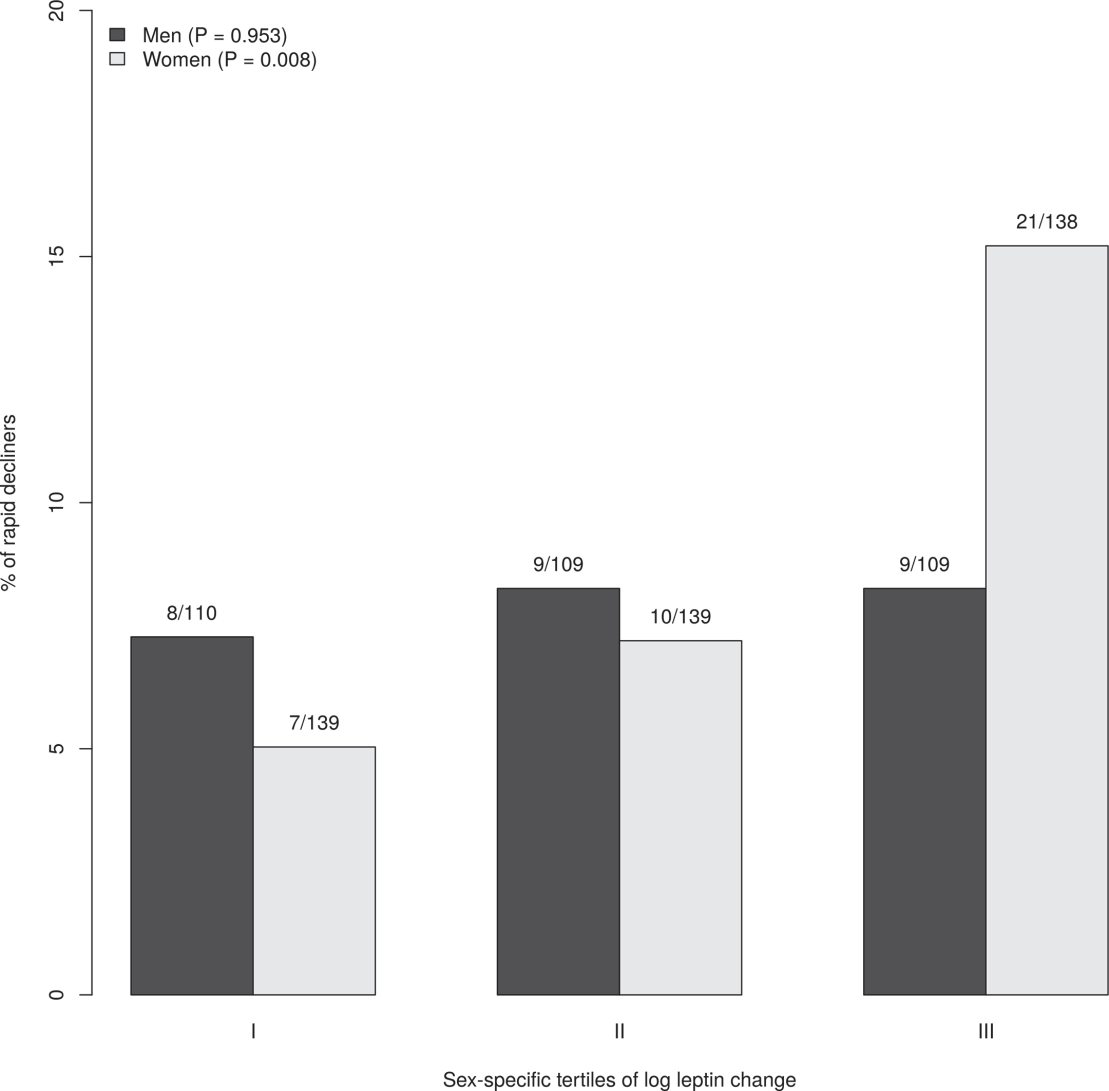
Proportion of “rapid decline” (eGFR decline > 3 ml/min/1.73 m) according to sex-specific tertiles of (log) leptin change.

The proportion of participants with a rapid decline of renal function was 8.6%, with higher figures in participants in the upper tertiles of leptin change over follow-up time (12.5% in the third tertile vs. 6% in the first tertile, P = 0.024). This association was not confirmed in men, while was more evident in women (Fig. 3).

## Discussion

This study provides the first demonstration of an association between increased serum leptin concentration and eGFR decline over time in a large cohort of community-dwelling older adults. Furthermore, in women there was an association between larger increase in serum leptin concentration and rapid kidney function decline.

Leptin has proinflammatory effects and promotes the synthesis of other cytokines such as TNF and IL-6 [26], that are also cleared by the kidney [33, 34]. Both IL-6 and TNF are increased in obese patients and have been associated with nephrotoxic effects in animal studies. TNF has been associated with podocytopathy [35], whereas IL-6 promoted renal damage in the context of the ischemia/reperfusion experimental model [36]. In our study, however, we found no association between these cytokines and decline of renal function in our study, suggesting that the experimental evidence pertaining to IL-6 and TNF does not apply to humans.

The association between leptin concentration and eGFR decline was evident in women but not in men. This finding may help to explain results from other studies [30, 31] showing a relationship between adiposity and renal failure in women but not in men. We found that leptin concentration was higher and more variable in women compared to men, and this finding does not seem to be related to body mass index or central adiposity. Further studies are needed to better explain the different behavior of leptin in the two sexes.

This is consistent with the fact that the vast majority of subjects in the third tertile of leptin were female. Males, instead, were more prevalent in the lowest tertile of leptin. As a consequence, a suggestion of an association between baseline leptin and eGFR decline was evident only in females.

Our findings confirm the complex association between fat tissue and renal impairment, and the difficulty of disentangling the effect of fat mass and leptin on the evolution of renal function. The data on this relationship are somewhat heterogeneous, with some studies pointing at the effect on intermediate factors, such as diabetes and hypertension [2, 3], and others at a direct nephrotoxic effect of obesity [7, 8, 37]. At any rate, weight loss is known to preserve renal function by a variety of mechanisms [38], and decreasing leptin may be one of these. Our findings suggest that classifying obese individuals on the basis of leptin serum levels may help to better characterize their risk for declining renal function.

Some limitations of this study are worth noting. We were unable to assess the direction of the association of higher leptin concentrations and impaired renal function. Leptin is partially cleared by the kidney, and thus our findings might to some extent reflect the effect of renal function on leptin concentration, rather than the contrary [39, 40]; the cited experimental research, however, supports a direct role of leptin. Furthermore, there may have been residual confounding due to imprecise measurements of adiposity. Visceral and not subcutaneous fat is known to account for the “inflammobesity” condition and might promote hypertension and, then, damage renal function also mechanically by directly compressing the kidney [41]. We used the waist to hip ratio as a measure of visceral obesity, but we cannot exclude that a more accurate index of fat distribution, e. g. a computed tomography estimate, might have weakened the role of fat-derived hormones like leptin. Indeed, obesity is initially associated with glomerular hyperfiltration and, then, increased GFR [9]. Our model included variables potentially lying in the causal pathway of the association between leptin and GFR. This leads more frequently to a conservative bias, as may be the case in this study. Indeed, in the sensitivity analysis excluding those variables from the model the effect of leptin on eGFR was more evident. Finally, we excluded a significant number of patients who did not participated in both follow-up visits; in particular those with the worst renal function were under-represented. Consequently, our data may not completely capture the relationship between leptin and eGFR among those with poor or very rapidly worsening renal function. This would suggest that our estimates may in fact be conservative.

In conclusion, present findings suggest that serum leptin may contribute to GFR decline independently of obesity and diabetes mellitus. Accordingly, a better characterization of adipokine profile of obese individuals may shed light on the accelerated renal function decline reported in a proportion of high-risk obese individuals. Finally, focusing on an earlier negative effect of leptin on the kidney, increased albuminuria [42], might disclose the nephrotoxic potential of leptin better than the assessment of the leptin-GFR relationship could.
